# Melatonin Induces Follicle Maturation in *Danio rerio*

**DOI:** 10.1371/journal.pone.0019978

**Published:** 2011-05-25

**Authors:** Oliana Carnevali, Giorgia Gioacchini, Francesca Maradonna, Ike Olivotto, Beatrice Migliarini

**Affiliations:** 1 Dipartimento di Scienze del Mare, Università Politecnica delle Marche, Ancona, Italy; 2 Istituto Nazionale Biostrutture e Biosistemi, Roma, Italy; New Mexico State University, United States of America

## Abstract

Most organisms modulate their reproductive activity responding to day length by the nocturnal release of melatonin by the pineal gland. This hormone is also responsible for synchronizing reproduction with specific external environment stimuli in order to optimize reproductive success.

The aim of this study was to establish the effect of melatonin on zebrafish reproduction.

Adult females were daily exposed, *via* water, to two different doses (100 nM and 1 µM) of melatonin. Melatonin led to an increase of the Gonado Somatic Index (GSI) associated with the increase of eggs production, and the raise of gene and protein levels of vitellogenin (VTG) and estradiol receptor α (ERα) in the liver. The ability of melatonin to increase fecundity was consistent with a significant increase of gene transcription of *kiss 1*, *kiss 2*, *gnrh3*, in the brain, and *lh* in the pituitary, while in the ovary (in class IIIB follicles), with a significant decrease of two genes codifying for intra-ovarian regulators of premature oocyte maturation, the *tgfβ1* and the *bmp15*. The reduction in the expression of these two genes was concomitant with the increase of *lhr* and a modulation of *mprα* and *mprβ* gene transcription, whose proteins are involved in oocyte maturation. Melatonin also exerted a direct action on follicles as shown by the increase of the oocytes undergoing to germinal vesicle break down (GVBD) and modulated *mpr α* and *β* gene expression in the *in vitro* exposure.

These data highlight the effects of melatonin in promoting zebrafish reproduction exerting its effects either in the brain-pituitary and in the gonads.

## Introduction

Melatonin, a multifunctional molecule, rhythmically secreted by the pineal gland, plays a major role in several circadian and seasonal processes including animal reproduction [1], [2], [3], [4], [5].

Reproduction is regulated by the neuroendocrine system: the reciprocal control of the brain and the endocrine system [6] contributes to adapting reproduction to environmental changes. In this regard, melatonin receptors have been found to be localized on the suprachiasmatic nuclei in the brain and in the pars tuberalis of the pituitary [7], [8] as well as in the ovary [9], [10] suggesting multiple melatonin sites of action in the modulation of reproduction.

The control of reproduction by melatonin is species-specific; in seasonal breeders, it is the duration of the daily melatonin secretion rather than its amplitude that encodes day length information [11], [12]. In addition, in mammals the administration of melatonin may induce or inhibit reproduction depending on the species reproductive strategy: melatonin induces a short day winter phenotype reproduction and inhibits long day summer phenotype [13], [14]. However, little information is currently available on the role of melatonin on zebrafish, a species that in laboratory conditions behaves as daily spawner with asynchronous ovaries [15].

The reproduction of seasonal and daily breeders involves a close association between the gonadotropin releasing hormone (GnRH) neurons and newly discovered kisspeptin fibers (metastin), [16], encoded by *kiss 1/kiss 2* genes. These genes, recently isolated also in zebrafish [17], were found to be present in the median eminence of primates [18], [19], in the arcuate nucleus (ARC) of rodents [20] and in the GnRH neurons of fish [21]. The presence of these neurons supports the role of KISS 1 [22] and especially of KISS 2 [16] as potential regulators of reproduction, including puberty. The kiss system is under photoperiod control and, depending on the species, melatonin may induce or inhibit the kiss 1 system and, in turn, the hypothalamic-pituitary-gonadal (HPG) axis. Evidence of the induction of reproduction following melatonin increase has been reported for the short day winter phenotype (i.e. sheep) [23] while inhibition occurs in the long day summer phenotype (i.e. hamsters) [14], [2], [20].

In all oviparous vertebrates, the oocytes arrest at prophase of the first meiotic division; during puberty or during annual recrudescence, the kiss system induces the HPG axis and oocytes enter vitellogenesis that characterizes oocyte growth. In fact, vitellogenic phase is under the neuroendocrine control of the GnRH secreted by the hypothalamus, stimulates gonadotropin release of both the follicle stimulating hormone (FSH) and the luteinizing hormone (LH). Gonadotropins, in turn, regulate gonadal steroidogenesis at theca and granulosa cell level, modulating steroidogenic enzymes that are essential for gamete maturation [24]. In females, estradiol promotes hepatic vitellogenin (*vtg*) gene transcription. This protein is secreted in the blood stream and, once in the ovary, it is selectively incorporated by a receptor mediated process and stored in the cytoplasm as yolk for further embryo development [25].

During the maturation phase, LH induces the production of the maturation inducing hormone (MIH) in the follicle and enhances the expression of the luteinizing hormone receptor (*lhr*) as well as two membrane progesterone receptors (*mprα* and *mprβ* isoforms) [26]. This pathway consists in the activation or, in some cases (as in fish), in the formation of the maturation promoting factor (MPF) made up of two subunits: cdc2 kinase and cyclin B. Subsequently, oocyte maturation occurs, the nuclear membrane is dissolved and germinal vesicle breakdown takes place (GVBD) [27].

In zebrafish, the locally produced growth factors such as insulin-like growth factors (IGFs), the transforming growth factor beta (TGFβ) super family including activins and bone morphogenetic proteins (BMPs) act in concert with gonadotropins to modulate follicular growth and prevent premature oocyte maturation by suppressing, in part, the sensitivity to MIH [28], [29].

Previous studies in mammals have shown that melatonin treatment remarkably increased the *lh* but not the *fsh* receptor mRNA transcription in granulosa cells [10] and a large number of scientific evidences supports the positive impact of melatonin on oocyte quality and fertilization rate [30], [31]. Despite increasing interest in the role of melatonin and its many physiological and pathological implications in mammals [32], there is still little information available regarding the role of melatonin in the endocrine control of reproduction at brain and gonadal level.

In this study, zebrafish was used in order to establish the role of melatonin in the neuroendocrine control of reproduction. Specifically, we investigated the biological effects of melatonin on *kiss 1* and *kiss 2* gene expression in the brain of females exposed to two different melatonin doses in relation to the expression of *gnrh3* and of *lh* in the pituitary.

Furthermore, the role of melatonin in zebrafish oocyte growth was tested by assaying the hepatic gene and protein levels of both VTG and estrogen receptor α (ERα). In addition, in order to test the possible influence of melatonin on the oocyte maturation process, the changes in *lhr*, *mprα*, *mprβ*, *bmp15* and *tgfβ1* gene expression were studied in class IIIB competent oocytes [33], [34]. Finally, the effects of exogenous melatonin administration on the maturation of competent class IIIB follicles were studied by *in vitro* exposure to two different doses of melatonin alone or in combination with the MIH, testing the induction of the GVBD and the expression of the following genes: *lhr*, *mprα*, *mprβ*, *bmp15* and *tgfβ1*.

The results of this study show for the first time a positive role of melatonin in the reproduction of a tropical species, the zebrafish *D. rerio*, providing the cascade of endocrine signals involved in the control of this process and in the direct action of melatonin on the ovarian follicles maturation leading to an increase of fecundity.

## Materials and Methods

### Ethics Statement

Procedures were performed in accordance with the Guidelines on the handling and Training of Laboratory Animals by the Universities Federation for Animal Welfare (UFAW) and with the Italian animal welfare legislation (D.L. 116/92).

### Animals drug exposure

Adult zebrafish females were purchased from a commercial dealer (Acquario di Bologna, BO, IT) and kept in aquaria at 28°C and oxygenated water. Fish were fed twice daily with commercial food (Vipagran, Sera, Germany) and other two times with *Artemia salina*. Eggs laid by parental fish were kept and grown. Six months old adult zebrafish were used for this study. Females were kept at 25±0,5°C, 14L [400 lux]/10D [1 lux] (light on at 7 a.m.) and divided in three groups (n = 13–15 each), one control and two groups were exposed to two different doses of melatonin (final concentration 100 nM and 1 µM) (Sigma, Milan, Italy), following Zhdanova and coworkers [35] and Piccinetti and coworkers [36] for 10 days *via* water in a semi-static condition. Treatment was 10 days long, melatonin doses were added daily at 11.00 a.m. and the concentrations were maintained constant throughout the experiment by renewing water every 24 hours in each tank. At the end of the exposure, five females from each group were paired with 2 control males to assay fecundity; the remaining females were sacrificed by a lethal overdose of anaesthesia (500 mg/L MS-222 [3-aminobenzoic acid ethyl ester] buffered to pH 7.4; Sigma). All fish were measured for total wet weight (mg) and ovary weight (to the nearest 0.1 mg) and the gonadosomatic index (GSI), derived by expressing the gonad weight as a percentage of the total body weight, was recorded.

Liver, ovary and brain including pituitary, were excided and immediately poured in liquid nitrogen and stored at −80°C until analysis were performed. The whole experiment was repeated two times.

### High-performance liquid chromatography

The melatonin levels in the tanks were measured after 30 min post addition by high-performance liquid chromatography (HPLC-FL) with fluorescence detection. HPLC assay was performed with a Beckman modular system (Beckman Instruments, San Ramon, CA, USA) with spectrofluorometric detector RF-551 (Shimadzu, Columbia, MD, USA), Chromatographic separations were carried out on an Ultrasphere C_18_ column (i.d., 250×4–6 mm; particle diameter, 5 µm; pore size, 80 Å). Excitation and emission wavelengths were set at 286 and 352 nm, respectively. An isocratic elution system was prepared. The mobile phase was 60% HPLC-grade methanol [37].

### Follicles collection

Fish were sacrificed as described above. Ovaries were excised from females, rinsed, and placed into sterile L15 medium at room temperature [33]. The ovaries were dissected into separate follicles using transfer pipettes (Samco Scientific Corp., San Fernando, CA) without trypsinization. Therefore, follicles were separated into different maturation stages according to diameters measuring with an ocular micrometer under a dissecting microscope and stage IIIB oocyte [38] were sampled for molecular, biochemical and *in vitro* maturation assays [39].

### Follicles in vitro maturation assay

*In vitro* maturation assays were conducted as previously described [29]. Briefly, stage IIIB follicles (0.52–0.69 mm diameter) were incubated in 1 ml medium at 25°C in 24-well culture plates. For experimental purpose, incubations were carried out separately in L15 (control), melatonin 50 pg/ml (MEL50), melatonin 100 pg/ml (MEL100), only MIH (1 µg/ml, MIH), melatonin 50 pg/ml (added 4 h before addition of 1 µg/ml MIH) and MIH (MEL50 + MIH) and melatonin 100 pg/ml (administered 4 h before addition of 1 µg/ml MIH) and MIH (MEL100 +MIH). The MIH here used was 17α, 20β dihydroxyprogesterone (100 ng/ml, Sigma Aldrich, Milan Italy). Maturation was scored after 12 hours of incubation. Follicles that underwent germinal vesicle breakdown (GVBD) could be identified by their acquired translucency. Each treatment was conducted in four wells with approximately 20 follicles per well, and all experiments were repeated three times. At the end of the experiments the follicles were stored at −80°C till molecular analysis.

### RNA extraction and cDNA synthesis

Total RNA from all livers, brains and class IIIB follicles mechanically isolated, was extracted with TRIREAGENT (Sigma) from all experimental groups. Final RNA concentrations were determined by optical density measurement at 260 nm, and the RNA integrity was verified by ethidium bromide staining of 28S and 18S ribosomal RNA bands on 1% agarose gel.

First strand cDNA synthesis was performed as already described in [40].

### Real time PCR

Triplicate PCR reactions were carried out for each sample analyzed. After real-Time condition optimization, PCRs were performed with SYBR green method in a iQ5 iCycler thermal cycler (Biorad). The reactions were set on a 96-well plate by mixing, for each sample, 1 µl of diluted (1/20) cDNA, 5 µl of 2× concentrated iQTM SYBR Green Supermix (Bio-rad), containing SYBR Green as a fluorescent intercalating agent, 0.3 µM forward primer and 0.3 µM of reverse primer. The primer sequences are shown in Table 1. The thermal profile for all reactions was 15′. at 95°C and then 45 cycles of 20″ at 95°C, 20″ at 60°C and 20″ at 72°C. Fluorescence monitoring occurred at the end of each cycle. Additional dissociation curve analysis was performed and showed in all cases one single peak.

**Table 1:** List of primers used for real time PCR analyses.

GENE	FOR PRIMER	REW PRIMER	AMPLICON
GnRH3	TTAGCATGGAGTGGAAAGGAAGGTTG	CTTTCAGAGGCAAACCTTCAGCAT	255
Kiss1	ACAGACACTCGTCCCACAGATG	CAATCGTGTGAGCATGTCCTG	131
Kiss2	ATTCTCTTCATGTCTGCAATGGTCA	TGCTTTCTCAGGTAAAGCATCATTG	154
LH	AATGCCTGGTGTTTCAGACC	AGTATGCGGGGAAATCCTCT	288
TGFβ1	TCGCTTTGTCTCCAAGGACT	TGCAAGAGAGTTGCCATTTG	234
BMP15	AGGGTGACCGGATCACTATG	TGCTGCCAGACTTTTTAGACC	291
LHR	CATTAACCTGCCCGACTGTT	AGTATGCGGGGAAATCCTCT	164
mPRα	CGGTTGTGATGGAGCAGATT	AGTAGCGCCAGTTCTGGTCA	185
mPRβ	CAACGAGCTGCTGAATGTGT	GGGCCAGTTCAGAGTGAGAC	197
ACT	AAAGTCCTCGCTGGAGACAA	TGCGAGCTGTGTGTGTATGA	242

Primers sequences were designed using Primer3 (v. 0.4.0) software (Accession numbers: GNRH3: NM_182887.2; KISS1: NM_001113489.1; KISS2: NM_001142585.1; LH: NM_205622.2; TGFβ1: NM_182873; BMP15: NM_001020484; LHR NM_205625.1; MPRα: NM_183345; MPRβ: NM_183344; ACT: NM_001168286.1).

The β-actin was used as housekeeping gene in each sample in order to standardize the results by eliminating variation in mRNA and cDNA quantity and quality. No amplification product was observed in negative control and no primers-dimer formation was observed in the control templates. The data obtained were analyzed using the iQ5 optical system software version 2.0 (Bio-rad). Modification of gene expression is represented with respect to the control.

### Western Blot analysis

For the VTG and ERα assays, liver samples were electrophoresed and transferred into PVDF as previously described in [40]. Briefly, 20 µg of each protein samples were separated using 4% stacking and 10% separating sodium dodecyl sulphate polyacrylamide gel electrophoresis (SDS-PAGE) as described by [41], and electroblotted onto a Bio-Rad filter using a Bio-Rad mini trans-blot electrophoretic transfer cell. The transfer was carried out for 2 hours at 250 mA 4°C using a 25 mM Tris base, 192 mM glycine, and 20% methanol as electrode solution. The membrane was soaked in 5% Nonidet-P40 for 1 h to remove SDS and incubated with 2% bovine serum albumin (BSA; Sigma) in PBS buffer. The vtg primary antibody (anti-vtg *D.rerio*, (Biosense Labs) was diluted 1∶1000 in a solution containing 2% BSA, 0.01% NaN3 in PBS and was incubated for 2 h at room temperature (about 20°C) and rinsed 3 times with PBS plus 0.05% Tween 20. For the ERα assay, the primary antibody (anti–estrogen Receptor 154–171), diluted 1∶1000 was purchased from Sigma. Anti-β-tubulin antibody (l g/mL) (Gene Tex, Inc.) was used to normalize the sample loading. The antibody reaction was visualized with ECL-PLUS (GE Healthcare) chemiluminescent reagent for Western blot.

The densitometric analysis was performed by ImageJ software for Windows.

### Statistical analysis

Real time and Western blotting results were examined by one-way ANOVA followed by the Bonferroni's multiple comparison test, GVBD rate raw data were firstly arcsin transformed and then analyzed, using a statistical software package, Prism5 (Graphpad Software, Inc. USA) with significance set at P<0.05.

## Results

### Melatonin assay

Melatonin concentrations in the tanks, found after 30 min. post addition, were comparable to nominal ones and reach the 50% of initial levels after 24 hrs.

### Effects of melatonin in the brain and pituitary

**Figure 1:**
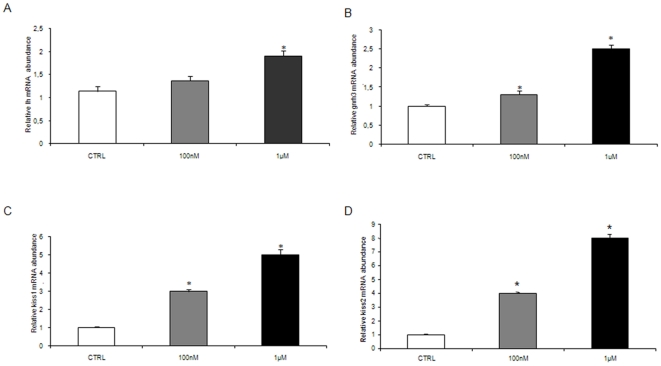
Effects of melatonin in the brain and pituitary. (A) *kiss1*, (B) *kiss2* (C) *gnrh3* and (D) *lh* mRNA levels, normalized against *β-actin* gene in fish exposed to 100 nM and 1 µM MEL, n = 15 per group. Error bars indicate mean±S.D. Asterisks denote exposure groups that are significantly different from the relative control group (CTRL) (p<0.05), analyzed using ANOVA followed by Bonferroni's multiple comparison test.

The endocrine control of reproduction by exogenous melatonin was demonstrated by the modulation of the hypothalamic genes involved. Melatonin administration significantly increased, in a dose related manner, the *gnrh3*, *kiss 1* and *kiss 2* gene transcription, as reported in Fig. 1. The *lh* gene transcription was increased by both melatonin doses but only the higher ones induced a significant increase (Fig. 1).

### Effects of melatonin on follicle growth

**Figure 2:**
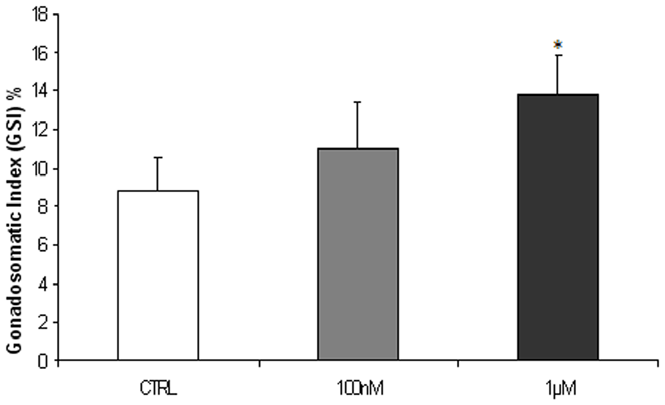
Effects of melatonin on GSI. Effect of MEL 100 nM and 1 µM on gonado- somatic index. n = 15 per group. Error bars indicate mean ± S.D. Asterisks indicate statistical significant differences respect to control group (P<0.05).

**Figure 3:**
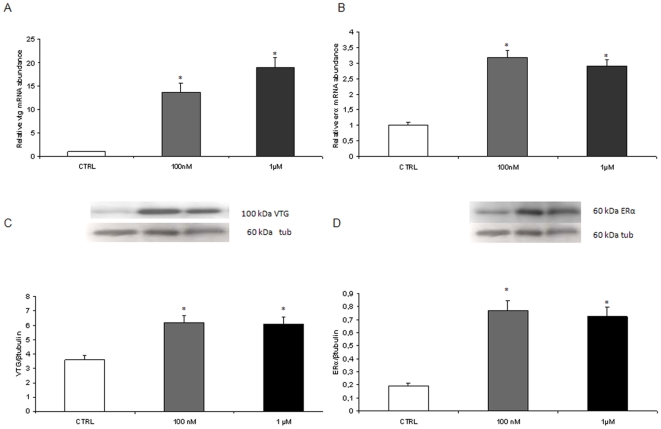
Effects of melatonin on follicles growth. (A) *vtg* and (B) *erα* mRNA levels, normalized against *β-actin* gene (C) VTG and (D) ERα typical Western blot normalized against β-TUBULIN and relative densitometric analysis, in liver of fish exposed to 100 nM and 1 µM MEL and control ones. n = 15 per group. Error bars indicate the mean±SD. Asterisks denote exposure groups that are significantly different from the relative control group (p<0.05), analyzed using ANOVA followed by Bonferroni's multiple comparison test.

These results show that complex ovarian follicle development can be modulated by exogenous melatonin, since GSI was significantly higher in fish exposed to the higher dose of melatonin (Fig. 2). A significant increase in both VTG and ERα gene expression and protein synthesis was found in the liver of fish treated with melatonin. The increase was similar in both doses used (Fig. 3A–D).

### In vivo effects of melatonin on follicle maturation

**Figure 4:**
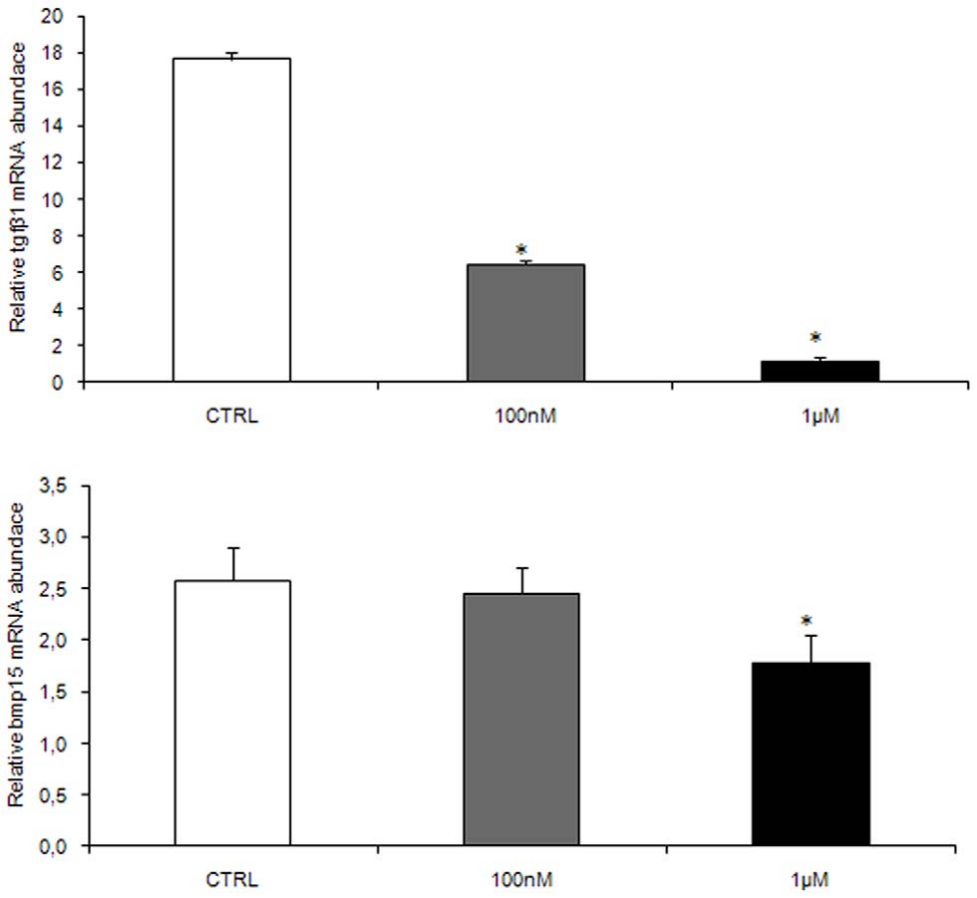
*In vivo* effects of melatonin on factors preventing precocious follicle maturation. (A) *tgfβ1* and (B) *bmp15* mRNA levels, normalized against *β-actin* gene in fish exposed to 100 nM and 1 µM MEL. n = 15 per group Error bars indicate mean±S.D. Asterisks denote exposure groups that are significantly different from the relative control group (CTRL) (p<0.05), analyzed using ANOVA followed by Bonferroni's multiple comparison test.

At molecular level, in class IIIB competent follicles the modulation of mRNA expression encoding for *lhr*, *mprα* and *β*, and of two local regulatory factors, *tgfβ1* and *bmp15* was demonstrated. With regards to the expression of the locally produced factors, *tgfβ1*gene expression was significantly reduced by both doses of hormone, while *bmp15* gene expression was significantly reduced only by the higher dose of melatonin (Fig. 4A,B).

**Figure 5:**
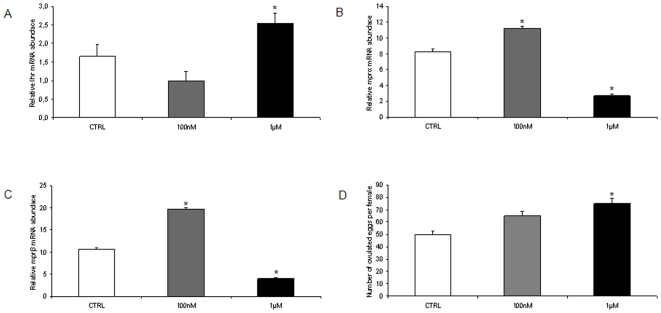
*In vivo* effects of melatonin on follicle maturation factors. (A) *lhr*, (B) *mprα* and (C) *mprβ* mRNA levels, normalized against *β-actin* gene in fish exposed to 100 nM and 1 µM MEL. n = 20 oocytes per group. Error bars indicate mean±S.D. (D) Number of ovulated eggs per female in fish exposed to 100 nM and 1 µM MEL. Asterisks denote exposure groups that are significantly different from the relative control group (CTRL) (p<0.05), analyzed using ANOVA followed by Bonferroni's multiple comparison test.

The higher dose of melatonin also increased *lhr* gene transcription (Fig. 5A). In regard to *mprα* and *mprβ* gene expression, the lower dose induced both isoforms; surprisingly the higher dose of melatonin resulted in a significant decrease of their expression (Fig. 5B,C).

In addition, melatonin significantly affected the fecundity. Higher numbers of ovulated eggs were observed in females exposed for 10 days to both doses of melatonin, when paired with control males. The increase was statistically significant (p<0.05) only with the higher melatonin dose (Fig. 5D).

### In vitro effects of melatonin on follicle maturation

**Figure 6:**
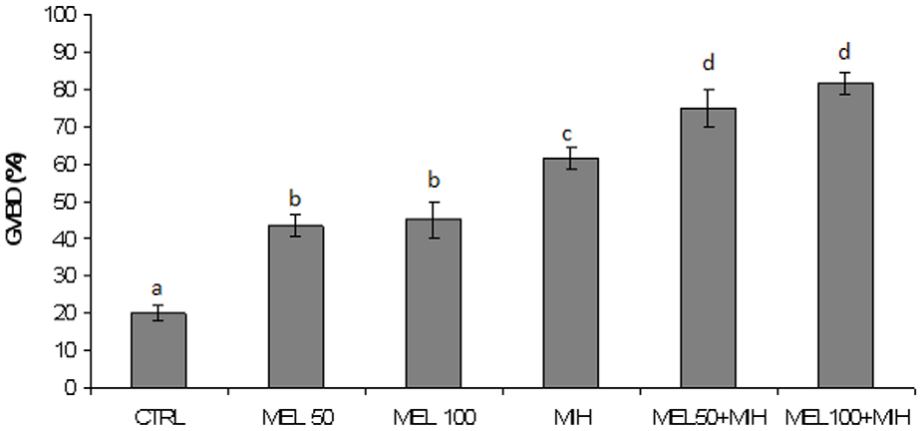
*In vitro* effects of melatonin on oocyte maturation. GVBD rate in class IIIB oocyte exposed to 50 and 100 pg/ml of melatonin alone or in combination with the MIH. Error bars indicate the raw data mean±SD. Different letters indicate statistically significant differences (P<0.05), after transformation of raw data in arcosin, analyzed by using ANOVA followed by Bonferroni's multiple comparison test.

The direct control of melatonin on follicle maturation was demonstrated by *in vitro* studies. The exposure of class IIIB follicles to 50 and 100 pg/ml of melatonin, alone or in combination with the MIH, induced follicle maturation. The greatest GVBD induction was obtained with the higher dose of melatonin plus MIH (Fig. 6).

**Figure 7:**
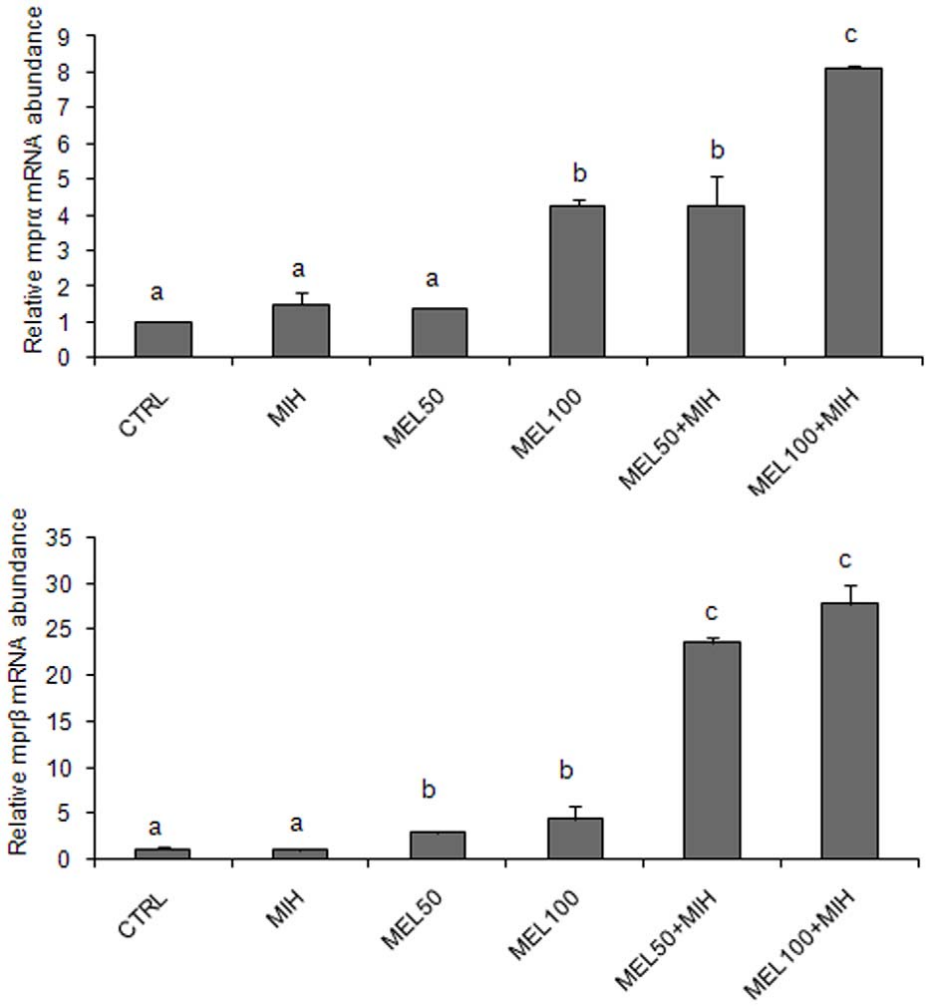
*mprα* and *mprβ* m RNA levels in class IIIB follicle. (A) *mprα* and (B) *mprβ* m RNA levels, normalized against *β-actin* gene in class IIIB follicle after exposure to 50 and 100 pg/ml of MEL, alone or in combination with the MIH. The different letters indicate statistically significant differences (P<0.05), analyzed using ANOVA followed by Bonferroni's multiple comparison test.

Melatonin *in vitro* treatment affected only the gene expression levels of the two *mpr* isoforms. The levels of these genes were significantly induced in follicles incubated with melatonin alone (both doses) and in higher extent with melatonin plus MIH (Fig. 7). On the contrary, no statistically significant differences were found regarding the expression levels of *lhr*, *tgfβ1* and *bmp15* respect to the control (data not shown).

## Discussion

Conflicting conclusions on the role of melatonin in the neuroendocrine system of teleost have been achieved by experiments dealing with photoperiod manipulation, pinealectomy and melatonin treatment [42], [43], [44], [4]. In this regard, in the eel, the implant of melatonin induced a decrease in LHβ and FSHβ [45] while, an increase in LHβ secretion was found in croaker [46], in sea bass [47] and in cultured carp pituitary [42], showing the ability of this hormone to affect reproduction.

In this study, melatonin induced a significant increase in zebrafish fecundity associated with changes in the endocrine and locally produced growth factors involved in oocyte growth and maturation.

The mechanism by which melatonin acts on the reproductive axis is still unknown although this hormone does not appear to have a direct influence on GnRH neurons [2] suggesting an upstream regulation of GnRH secretion. In fact, in mammals, kisspeptins have been shown to be integral in the regulation of GnRH modulation and the levels of melatonin are correlated both to a decrease in kiss 1 in the ARC of the Syrian hamster, a long day breeder, and to an increase in the sheep, a short day seasonal breeder. These evidences indicate a role of melatonin in the photoperiodic control of reproduction through the modulation of HPG axis by kiss neurons (reviewed in [2]). This effect was recently confirmed in ewe by Chalivoix and coworkers [48]. In mammals, the kiss system is currently thought to be the link mediating the response between environmental cues and metabolic signals and the key system in the regulation of puberty and reproduction [49].

Surprisingly, the results obtained in this study suggest that melatonin might act as a signal mechanism switching for reproduction in zebrafish, by activating the cascade starting from the kiss peptide which, in turn, stimulates hypothalamic neurons to produce GnRH.

In this study, melatonin was provided once a day, at the same time for ten days to cover the entire oocyte maturation cycle, that in zebrafish has been determined as occurring in eight days [50], [27].

The concomitant increase in GSI and VTG synthesis in the liver, observed in these females, supports the positive role of melatonin in oocyte growth and suggests its involvement in the vitellogenic process. These results are in agreement with the increase of vitellogenic follicles found in *Channa punctatus* exposed to melatonin *via* water for 24 h [5] but apparently in contrast with those previously obtained in trout, a typical seasonal breeder, in which the release of melatonin is not regulated by the internal clock [51], as on the contrary occurs in zebrafish [52]. In trout, no differences in *vtg* and *erα* gene expression levels were observed in hepatocytes exposed to melatonin *in vitro*. A slight but significant stimulatory effect on *er* expression was observed in the liver of females implanted with melatonin, although this effect had no impact on *vtg* gene expression [53].

The effect on vitellogenin synthesis in zebrafish liver, might be the consequence of the melatonin ability to activate the HPG axis, as suggested by the significant increase in the brain of *kiss 1* and *kiss 2* gene expression. In tilapia, the *kiss 1 receptor* is expressed in the GnRH neurons [21]. In fathead minnow, KISS1 stimulates the synthesis of gnrh3, one of the most hierarchically important hypothalamic signals, and induces the pituitary to produce gonadotropins responsible for the activation of gonadal steroidogenesis, including the increase in estradiol and in the different isoforms of its receptor [54]. In this study, the increase in *kiss* gene levels appears to be related to *gnrh3* gene expression.

Previous results in mammals evidenced the positive effects of LEPTIN on *kiss 1* [49]. The increase of *kiss 1* and *2* gene expression found in our study may be related with the high levels of *leptin* [36] previously determined in our laboratory in the brain of zebrafish exposed to the same melatonin doses at the same conditions used in this study. The concomitant increase in *kiss 1*, *kiss 2*, *gnrh3* and *lh* gene expression found in the present study, agrees with the increase observed in mammalian short day seasonal breeders [48] and clearly indicates an endocrine control of melatonin on zebrafish reproduction. The activation of the HPG axis by melatonin is confirmed by the increase in GSI and eggs production. On the contrary, in a previous study conducted in eel implanted with melatonin, the effects of this chronic treatment showed that melatonin had no effect on mGnRH and cGnRH-II, and that the continuous release of melatonin stimulates the dopaminergic system of the preoptic area with a consequent inhibitory control of gonadotropin, deeply affecting reproduction [45]. The different effects observed in the melatonin implanted eel and the zebrafish females exposed to melatonin *via* water may be related either to the different route of exposure or to their significantly different reproductive strategy.

In this study, in addition to melatonin action at liver, brain and pituitary level, the effect of this hormone was also shown in the ovary. In class IIIB competent follicles, from *in vivo* exposed females to melatonin, induction of signals involved in final oocyte maturation such as *lhr* and *mprs* (only with lower dose) was found. These increases were associated with the down-regulation of two local factors: *tgfβ1* and *bmp15* genes. These two intra-ovarian regulators of follicle development act at multiple sites. Previous studies have shown that the inhibiting effects of *tgfβ1* are in part due to the regulation of gene transcription including *lhr* and *mprα* and *mprβ* [55] and also appear to be involved in the regulation of the *erα* [56] that in mammals has a specific role in ovulation [57].

The down-regulation of *mprs* we found by *in vivo* study in class IIIB follicles with the higher dose of melatonin opens up several questions on the involvement of this hormone in the acquiring of oocyte competence as previously observed in mammals [58]. In addition, the control of melatonin on *mprs* by *in vitro* study demonstrated a direct effect of melatonin on the transcription of very important signals involved in oocyte competence and maturation, suggesting a local control of this hormone beside the endocrine ones.The direct role of melatonin in the ovary is supported by the presence of a melatonin receptor (MEL 1A1) in the membrane of class IIIB oocyte in carp [59] and by the *in vitro* study. Chattoraj and co-workers [60] through *in vitro* studies, demonstrated that melatonin, added to the medium 4 hrs before the addition of MIH, accelerated GVBD rate in denuded oocytes of two carp species. In these carp, the efficacy of the melatonin dose was species specific. Recently the same authors [61] demonstrated the direct action of melatonin in the regulation of meiotic resumption in carp oocytes. In zebrafish, we also found a direct effect of melatonin on *in vitro* follicle maturation supporting its role at local level.

In addition, an induction of *leptin* gene transcription by melatonin was previously observed in the zebrafish ovary [62] suggesting that the positive role exerted by melatonin in zebrafish reproduction may also involve LEPTIN at gonadal level as suggested by Moschos and co-Workers [63].

The large variability of the impact of melatonin on brain, pituitary and on gonadal factors observed among vertebrates indicates species-specific responses to this hormone.

Additional studies using different methodological approach such as the Fourier Transform Infrared (FTIR) microspectroscopy, a powerful method to study the composition and the macromolecular chemistry of fish oocytes [64], [65] are actually in progress to provide e a biochemical fingerprint of oocytes from females exposed to melatonin to better understand the role of this hormone on zebrafish reproduction.
